# SSRMMD: A Rapid and Accurate Algorithm for Mining SSR Feature Loci and Candidate Polymorphic SSRs Based on Assembled Sequences

**DOI:** 10.3389/fgene.2020.00706

**Published:** 2020-07-27

**Authors:** Xiangjian Gou, Haoran Shi, Shifan Yu, Zhiqiang Wang, Caixia Li, Shihang Liu, Jian Ma, Guangdeng Chen, Tao Liu, Yaxi Liu

**Affiliations:** ^1^Triticeae Research Institute, Sichuan Agricultural University, Chengdu, China; ^2^Maize Research Institute, Sichuan Agricultural University, Chengdu, China; ^3^College of Resources, Sichuan Agricultural University, Chengdu, China; ^4^College of Information Engineering, Sichuan Agricultural University, Ya’an, China; ^5^State Key Laboratory of Crop Gene Exploration and Utilization in Southwest China, Chengdu, China

**Keywords:** bioinformatics, algorithm, simple sequence repeats, conservativeness, uniqueness, polymorphism

## Abstract

Microsatellites or simple sequence repeats (SSRs) are short tandem repeats of DNA widespread in genomes and transcriptomes of diverse organisms and are used in various genetic studies. Few software programs that mine SSRs can be further used to mine polymorphic SSRs, and these programs have poor portability, have slow computational speed, are highly dependent on other programs, and have low marker development rates. In this study, we develop an algorithm named Simple Sequence Repeat Molecular Marker Developer (SSRMMD), which uses improved regular expressions to rapidly and exhaustively mine perfect SSR loci from any size of assembled sequence. To mine polymorphic SSRs, SSRMMD uses a novel three-stage method to assess the conservativeness of SSR flanking sequences and then uses the sliding window method to fragment each assembled sequence to assess its uniqueness. Furthermore, molecular biology assays support the polymorphic SSRs identified by SSRMMD. SSRMMD is implemented using the Perl programming language and can be downloaded from https://github.com/GouXiangJian/SSRMMD.

## Introduction

Owing to their abundance, codominant inheritance, multi-allelic nature, transferability, and ease of analysis *via* PCR (Varshney et al., 2005; Ramu et al., 2009; Kaur et al., 2015), simple sequence repeat (SSR) markers have been successfully adopted in various genetic studies such as quantitative trait loci mapping (Qin et al., 2015; Wang et al., 2017), genotyping (Gramazio et al., 2018), genetic diversity (Nachimuthu et al., 2015; Zhou R. et al., 2015), and DNA fingerprinting (Zhang et al., 2015). Indeed, numerous genome-wide SSR markers have been identified in plants and animals in recent years, such as those in rice (Zhang et al., 2007), maize (Xu et al., 2013), cucumber (Liu et al., 2015), bee (Liu et al., 2016), tobacco (Wang et al., 2018), and snake (Liu et al., 2019).

During the development of SSR markers, the first step is the mining of potential SSR loci from assembled sequences. Based on the repetitive architecture of their motifs, SSRs can be classified as perfect (e.g., AGAGAGAGAGAG), and imperfect (including nucleotide substitutions or indels, e.g., AGAGAGACAGAG). However, the application of perfect SSRs in genetic studies far exceeds that of imperfect SSRs because of its higher allelic variability (Zalapa et al., 2012; Xu et al., 2013). Numerous algorithms and software programs have been reported for mining perfect SSRs. For instance, SSRIT (Temnykh, 2001), MISA (Thiel et al., 2003), and GMATo (Wang et al., 2013) use regular expressions based on the greedy matching algorithm to mine SSRs. SA-SSR (Pickett et al., 2016) uses a suffix array-based algorithm to mine SSRs. Kmer-SSR (Pickett et al., 2017) uses Kmer decomposition to identify SSRs. PERF (Avvaru et al., 2017) matches each potential substring in accordance with a set of pre-computed repeat strings. Other programs including TROLL (Castelo et al., 2002), MfSAT (Chen et al., 2011), ProGeRF (Silva et al., 2015), and FullSSR (Metz et al., 2016) have also been developed. In addition, imperfect SSR detection algorithms have also been reported, such as IMEx (Mudunuri and Nagarajaram, 2007), and Krait (Du et al., 2017). However, these programs have many common undesirable features. First, they rely on additional software or modules, often with complex software configuration; second, they have poor portability and can only be run on Linux or Windows platforms; third, they have slow computational speed; and most importantly, polymorphic SSRs cannot be directly found.

With rapid advancements in genomics, software and pipelines for mining polymorphic SSRs have been reported. For instance, CandiSSR (Xia et al., 2016), a candidate polymorphic SSRs identification pipeline, is based on multiple assembly sequences. GMATA (Wang and Wang, 2016) provides a complete process for SSR markers development. IDSSR (Guang et al., 2019) has recently been reported to identify polymorphic SSRs in a single genome sequences using a similar pipeline. However, these programs or pipelines also share certain issues. First, they rely on numerous other programs, such as MISA (Thiel et al., 2003), Primer3 (Untergasser et al., 2012), BLAST (Altschul et al., 1997), and ClustalW (Thompson et al., 2002); second, they have slow computational speed for mining polymorphic SSRs; finally, they have low rates of SSR markers development.

To overcome these limitations, we developed the Simple Sequence Repeat Molecular Marker Developer (SSRMMD) program using the Perl programming language. This program rapidly and exhaustively mines perfect SSR loci through improved regular expressions. For mining polymorphic SSRs, this program uses a high-stringency sequence alignment algorithm to assess the conservativeness and uniqueness of SSR flanking sequences. Compared with other software programs, SSRMMD is more rapid, accurate, and convenient. SSRMMD can be downloaded from https://github.com/GouXiangJian/SSRMMD.

## Materials and Methods

### Implemented Algorithm

**FIGURE 1:**
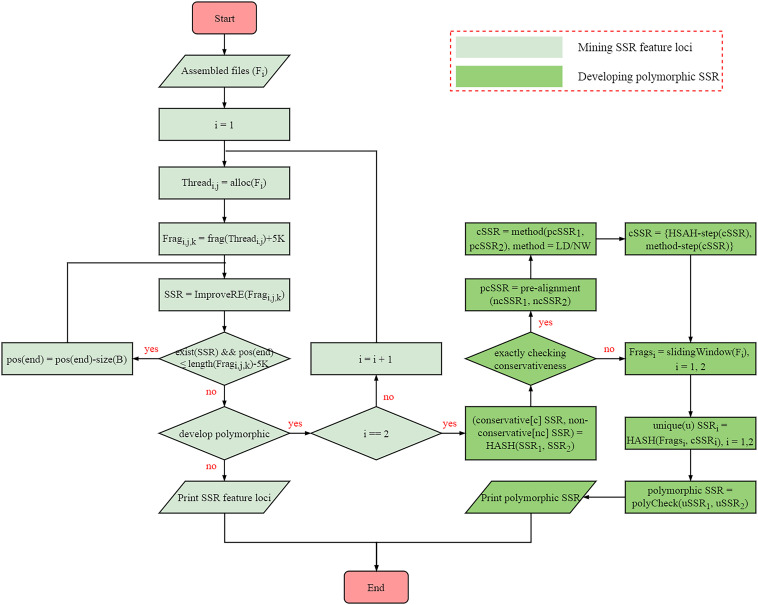
Internal implementation algorithm of Simple Sequence Repeat Molecular Marker Developer (SSRMMD).

The algorithm of SSRMMD involves the mining of perfect SSR loci and the discovery of polymorphic SSRs. The internal methodological details are provided in Figure 1, primarily including the following steps:

(1) Mining perfect SSR loci. Similar to programs such as SSRIT (Temnykh, 2001) and MISA (Thiel et al., 2003), SSRMMD uses regular expressions with the greedy matching algorithm to mine SSRs. However, to improve computational speed, SSRMMD was optimized in three aspects: (i) use of multi-threading technology. To maximize the function of each thread, we proposed a novel optimal allocation algorithm to averagely distribute assembled sequences to each thread in accordance with the length of sequences (TOS), including the following: (a) sort sequences by TOS; (b) assignment of the longest *i* sequences to *i* threads; (c) thread sorting based on the total TOS; (d) assignment of subsequent sequences to the thread with the smallest TOS; (e) thread sorting in step (d) using the insertion sorting algorithm; and (f) iterative performance of steps (d) and (e) until complete sequence allocation. (ii) Fragmented sequences. After a specific thread is assigned to store each sequence, SSRMMD fragmented each sequence into short 500-kb fragments. At this length, the computational speed was the highest. Furthermore, 5 kb was added to each fragment to prevent potential SSRs from being cut off. (iii) Improved regular expression. Ordinary regular expressions can only mine one type of motif in each match, as indicated using MISA (Thiel et al., 2003). However, by integrating all patterns, SSRMMD can mine all types of motif in each match, indicating that irrespective of the arrangement of the threshold motifs, SSRMMD will only traverse the sequence once. Notably, to completely mine compound SSRs, SSRMMD backtracks after each successful match, and the size of backtracking (B) is as follows:

$$s\,i\,z\,e\,\left(B\right)=\left\{ \begin{array}{c} length\left(motif_{i}\right)-1,sum\left(motif_{i}\right)==1\\ \max\left\{L_{1},L_{2},\ldots ,L_{n}\right\},\\ \text{min}\left\{S_{1},S_{2},\ldots ,S_{n}\right\}\;>\max\left\{L_{1},L_{2},\ldots ,L_{n}\right\}\\ \min\left\{S_{1},S_{2},\ldots ,S_{n}\right\}-1,\\ \min\left\{S_{1},S_{2},\ldots ,S_{n}\right\}\;\leq \max\left\{L_{1},L_{2},\ldots ,L_{n}\right\} \end{array}\right.$$

where *n* is the number of motif types, *S*_*i*_ is the length of the *i*th motif types of SSR, and *L*_*i*_ is the length of the *i*th type of motif.

(2) Assessment of the conservativeness of SSR flanking sequences. To develop polymorphic SSRs, we initially assessed the conservativeness of SSR flanking sequences. To maximize the computational speed, we used a novel three-stage method to align flanking sequences between two assembled sequences files, which included the following steps: (i) first, absolutely conserved flanking sequences were filtered out using HASH structure. Herein, we considered these flanking sequences in the first assembled file as a library, and then we compared these flanking sequences in the another assembled file with the aforementioned library to rapidly identify absolutely conserved flanking sequences. (ii) Second, conservativeness pre-alignment was performed using *x*% [default is 5% (each side is 5 bp)] flanking sequences near SSRs. Assuming that flanking sequences near SSRs were highly conserved, SSRMMD allowed flanking sequences near SSRs to tolerate up to 2-bp mismatches. Moreover, after extensive assessments, additional mismatches (≥3 bp) did not further benefit the results, consistent with the aforementioned assumption. SSRMMD iteratively replaced mismatched bases and aligned flanking sequences between two assembled files, using a method similar to (i). (iii) Finally, SSRMMD used Levenshtein distance (LD; Levenshtein, 1966), or the Needleman–Wunsch (NW) algorithm (Needleman and Wunsch, 1970) to accurately assess the conservativeness of the flanking sequences retained through pre-alignment. LD was defined as the minimum number of edits required to convert one string to another, thus indirectly reflecting the identity of two DNA sequences. However, the NW algorithm based on dynamic programming has been extensively used for global sequence alignment, directly reflecting the identity of two DNA sequences. Compared with NW algorithm, the LD did not require backtracking; hence, it had a higher computational speed; furthermore, the NW algorithm had a more comprehensive scoring system than LD, thus facilitating more accurate elucidation of the identity of the SSR flanking sequences. The iterative formulae of the LD and NW algorithms are as follows:

$$L\,D_{a,b}\,\left(i,j\right)=\left\{ \begin{array}{c} \max\left(i,j\right)\;\text{min}\,\left(i,j\right)=0\\ \min\,\left\{ \begin{array}{c} L\,D_{a,b}\,\left(i-1,j\right)+1\\ L\,D_{a,b}\,\left(i,j-1\right)+1\;o\,t\,h\,e\,r\,w\,i\,s\,e\\ L\,D_{a,b}\,\left(i-1,j-1\right)+1_{\left(a_{i}\neq {\text{b}}_{j}\right)} \end{array}\right. \end{array}\right.$$

$$N\,W_{a,b}\,\left(i,j\right)=\left\{ \begin{array}{c} 0\;\text{i},\text{j}=0\\ m\,a\,x\,\left\{ \begin{array}{c} N\,W_{a,b}\,\left(i-1,j\right)+\\ S_{g\,a\,p}\;a_{i}\,a\,l\,i\,g\,n\,e\,d\,t\,o\,a\,g\,a\,p\\ N\,W_{a,b}\,\left(i,j-1\right)+\\ S_{g\,a\,p}\;b_{j}\,a\,l\,i\,g\,n\,e\,d\,t\,o\,a\,g\,a\,p\\ N\,W_{a,b}\,\left(i-1,j-1\right)+\\ S_{m\,a\,t\,c\,h/m\,i\,s\,m\,a\,t\,c\,h}\;a_{i}\,a\,l\,i\,g\,n\,e\,d\,t\,o\,b_{j} \end{array}\right. \end{array}\right.$$

where *a* and *b* are 2 strings; *i* and *j* are subscripts of *a* and *b*, respectively; *S*_*match*_ is the score of match; *S*_*mismatch*_ is the score of mismatch; and *S*_*gap*_ is the score of gap.

(3) Assessment of the uniqueness of SSR flanking sequences. After conservativeness was assessed, SSRMMD further assessed the uniqueness of SSR flanking sequences. Again, assembled sequences were evenly distributed to each thread and were fragmented through the sliding window method, wherein window size was the length of flanking sequences, the step size was 1 bp, and all fragments were stored in a HASH database. Thereafter, flanking sequences with the equal sizes in the aforementioned HASH database were aligned to identify SSRs with unique flanking sequences. Finally, polymorphisms were compared in the two unique SSR sets to distinguish monomorphic and polymorphic SSRs. Notably, to meet different needs, SSRMMD used two computational methods, (i) running in a time-saving manner and (ii) running in a memory-saving manner, indicating that SSRMMD functions adequately, irrespective of the use of a personal computer, or high-performance server.

### Input and Output

Assembled sequences (e.g., genome, transcriptome, or a single gene) with a standard FASTA format is required for mining SSRs; to further develop candidate polymorphic SSRs, another assembled sequence is required. Certain parameters can be set to change the SSR mining conditions, including motif threshold and the length of flanking sequences. SSRMMD is allowed to set any size motif (>6 bp), and SSRMMD would then assess the conservativeness and uniqueness of SSR flanking sequences when mining polymorphic SSRs. Notably, setting more threads would significantly enhance the computational speed.

Upon completion of the computation, SSRMMD yields three types of outputs: (i) detailed information record file of SSRs; (ii) statistical file of SSRs, which analyzes the various distribution characteristics of SSRs and helps understand the distribution pattern of the SSRs [including the following: (a) SSR number and density in each assembled sequence; (b) SSR number and proportion per unit length of the motif; and (c) SSR number among different numbers of repeats in each motif]; and (iii) detailed information record file of candidate polymorphic SSRs.

### Performance Test Datasets

To assess SSRMMD, we downloaded six genomes of three plants from National Center for Biotechnology Information (NCBI)^1^ and Unité de Recherche Génomique Info (URGI)^2^. Three genomes were used to assess the potential for mining SSR feature loci, including rice (Zhenshan97, ∼0.39 Gb), cotton (TM1, ∼2.29 Gb), and wheat [Chinese Spring (CS), ∼14.23 Gb]. All six genomes were used to assess the potential to mine polymorphic SSRs, including two rice genomes, two cotton genomes, and two wheat genomes. To evaluate the complexity and multi-threading of SSRMMD, we extracted 2-Gb sequences from the wheat CS and AK58 genomes, which were evenly divided into 20 sequences. The GenBank assembly accession numbers of the rice genomes were Zhenshan97 (GCA_001623345.2) and Shuhui498 (GCA_002151415.1). The GenBank assembly accession numbers of cotton genomes were TM1 (GCA_006980745.1) and ZM24 (GCA_006980775.1). The wheat CS and AK58 genomes were obtained from URGI.

### Performance Test Parameters

Perfect repeats have higher allelic variability than imperfect repeats, and any SSR used to develop genetic markers should contain a perfect repeat (Xu et al., 2013). Therefore, to assess the potential of SSRMMD for mining SSR loci, we avoided imperfect repeats detection tools, and we selected six popular existing software programs including SSRIT (Temnykh, 2001), MISA (Thiel et al., 2003), GMATA (Wang and Wang, 2016), SA-SSR (Pickett et al., 2016), Kmer-SSR (Pickett et al., 2017), and PERF (Avvaru et al., 2017). In particular, SA-SSR was not included in the results owing to its markedly low computational speed. In each software program, based on previously described methods (Zhang et al., 2007; Xu et al., 2013; Liu et al., 2015), the minimum repeat times of SSR motif lengths of 1, 2, 3, 4, 5, and 6 bp were set to 10, 7, 6, 5, 4, and 4, respectively. Because Kmer-SSR can use multi-threads, we tested SSRMMD and Kmer-SSR with 1 and 12 threads, respectively, to assess its multi-thread support. However, other software programs could only use a single thread.

To assess the potential for mining polymorphic SSRs, we compared two popular existing software programs with SSRMMD, including GMATA (Wang and Wang, 2016), and CandiSSR (Xia et al., 2016). In each software program, SSR flanking sequences were set to 150 bp (Zhang et al., 2007). Because CandiSSR can use multi-threads, we assessed SSRMMD, and CandiSSR with 12 threads; however, GMATA can only use a single thread. On assessing SSRMMD, LD was used to assess the conservativeness of flanking sequences, and the threshold was set to 5% to correspond to the BLAST identity of CandiSSR, and the other parameters (not indicated herein) were retained as default setting. Similarly, parameters not included in GMATA and CandiSSR were used with default setting.

### Performance Evaluation Criteria

The performance of SSRMMD and existing software programs for mining perfect SSRs was evaluated in accordance with six criteria. Table 1 shows the portability, dependence, and function of existing software programs and SSRMMD. The computational accuracy, speed, and memory consumption were evaluated for the test datasets. We used the Linux *time* command to record the computational time and *pmap* command to record the memory peak. All tests were performed using a personal computer with an Intel^®^ Xeonl^®^ CPU E5-2683 v3 @ 2.00 GHz with CentOS Linux release 7.4.1708 and 64 GB RAM.

**TABLE 1:** Various features of SSRMMD and existing software programs for mining perfect SSRs.

Software	Year	Portability	Dependence^*a*^	Function
SSRIT	2001	Windows/Linux	No	Mining SSR feature loci
TROLL	2002	Windows/Linux	Staden	Mining SSR feature loci
MISA	2003	Windows/Linux	No	Mining SSR feature loci
MfSAT	2011	Windows	No	Mining SSR feature loci
GMATo	2013	Windows/Linux	No	Mining SSR feature loci
ProGeRF	2015	Linux	No	Mining SSR feature loci
CandiSSR	2016	Linux	MISA, BLAST, Primer3, Clustalw	Developing polymorphic SSRs
FullSSR	2016	Windows/Linux	BioPerl, Bio:Tools:Run: Primer3	Mining SSR feature loci
GMATA	2016	Windows/Linux	Primer3, e-PCR	Developing polymorphic SSRs
SA-SSR	2016	Linux	No	Mining SSR feature loci
Kmer-SSR	2017	Linux	No	Mining SSR feature loci
PERF	2017	Windows/Linux	tqdm, biopython	Mining SSR feature loci
IDSSR	2019	Linux	SSRIT, BLAST, Primer3	Developing polymorphic SSRs
SSRMMD	this	Windows/Linux	No	Developing polymorphic SSRs

*Note. SSRMMD, Simple Sequence Repeat Molecular Marker Developer. ^*a*^Dependence on additional programs or modules.*

### Experimental Validation

To verify the accuracy of the output through SSRMMD, 80 pairs of polymorphic SSRs were randomly selected from the computational results of wheat for molecular biology assays. These selected polymorphic SSRs were evenly distributed on each chromosome, encompassing differently sized motifs. Genomic DNA was extracted using the cetyl trimethylammonium bromide (CTAB) method from fresh leaves of 10 wheat popularized and local cultivars of CS, AK58, CM107, CN16, MM37, ZM012542, ZM000652, ZM018703, ZM003222, and ZM003284.

Additionally, we provided a tool named connectorToPrimer3 to associate SSRMMD with Primer3 (Untergasser et al., 2012); hence, a primer design can be easily performed. The primary parameters were as follows: (1) minimum, optimal, and maximum primer sizes of 18, 20, and 27 bp, respectively; (2) minimum and maximum GC contents of 20% and 80%, respectively; (3) minimum, optimal, and maximum Tm values of 57, 60, and 63°C, respectively; and (4) product lengths of 100–300 bp. Primers were synthesized by Beijing Qingke Biotechnology Co., Ltd.

PCR was performed in 10-μl reactions containing 5 μl of mix buffer (2×), 1.0 μl of template DNA (100 ng/μl), 0.5 μl of primers, and 3 μl of ddH_2_O. The PCR conditions were as follows: 1 cycle at 94°C for 5 min, 35 cycles at 94°C for 30 s, 60°C for 30 s, 72°C for 30 s, and 1 cycle at 72°C for 10 min. The PCR products were electrophoresed on a 6% denaturing polyacrylamide gel. SSR polymorphisms in different wheat genotypes were identified on the basis of differences in mobility, as revealed through the electrophoretic bands.

## Results

### Assessment of Complexity and Threads

**FIGURE 2:**
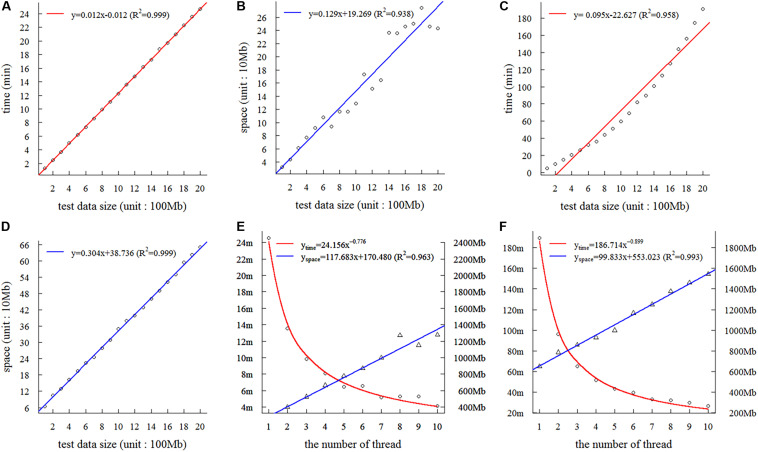
Assessment of complexity and threads of Simple Sequence Repeat Molecular Marker Developer (SSRMMD). **(A)** The time complexity in mining SSR feature loci. **(B)** The space complexity in mining simple sequence repeat (SSR) feature loci. **(C)** Time complexity in developing polymorphic SSRs. **(D)** The space complexity in developing polymorphic SSRs. **(E)** The impact on time and space when increasing threads in mining SSR feature loci. **(F)** The impact on time and space when increasing threads in developing polymorphic SSRs.

On the basis of the 2-Gb base sequences from wheat, we tested the time and space complexity of SSRMMD in a single thread. As shown in Figures 2A,B, as the amount of data increased, the time and space consumed by SSRMMD increased linearly when mining SSR feature loci. Similarly, when SSRMMD was used to mine polymorphic SSRs (assessing uniqueness in a memory-saving manner), the time and space were also linearly associated with the amount of data (Figures 2C,D). These results suggest that the algorithm of SSRMMD has linear time complexity [T(*n*) = O(*n*)] and space complexity [S(*n*) = O(*n*)].

Furthermore, we assessed the multi-threading support of SSRMMD. As shown in Figures 2E,F, whether mining in SSR feature loci or polymorphic SSRs, as the number of threads increased, time consumption decayed as a power function with the number of threads; however, memory consumption scaled linearly. Notably, despite using 10 threads, memory consumption of SSRMMD did not exceed the size of test data (2 Gb). In total, SSRMMD adequately supported multi-threading.

### Verification of the Performance of SSRMMD to Mine Simple Sequence Repeat Feature Loci

**FIGURE 3:**
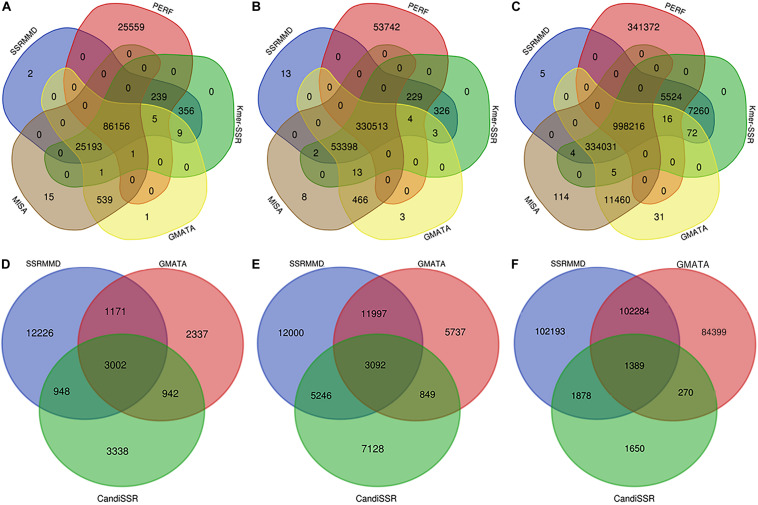
Overlapping simple sequence repeats (SSRs) calculated by five programs [Simple Sequence Repeat Molecular Marker Developer (SSRMMD), PERF, Kmer-SSR, GMATA, and MISA], and overlapping polymorphic SSRs calculated by three programs (SSRMMD, GMATA, and CandiSSR). If the sequence name, start, and end position were the same in all programs, the SSR was considered overlapping. **(A)** Rice. **(B)** Cotton. **(C)** Wheat. **(D)** Rice. **(E)** Cotton. **(F)** Wheat. Notably, CandiSSR only mined 5,187 polymorphic SSRs of wheat chromosome 5D.

Based on the high citation rate and novel principles, six software programs were compared with SSRMMD (SA-SSR is not indicated). As shown in Table 2, SSRMMD identified the most SSRs. This was larger than other regular expression-based programs including MISA and GMATA. Furthermore, SSRMMD had the highest computational speed when running on a single thread and better supported multi-threading than Kmer-SSR. Additionally, we analyzed the validity of SSRs found by SSRMMD and compared them with four other programs (PERF, Kmer-SSR, GMATA, and MISA). As shown in Figures 3A–C, numerous common products were identified in these software programs, accounting for 76.95% (rice), 85.96% (cotton), and 74.21% (wheat) of SSRMMD, respectively.

**TABLE 2:** Performance comparison between SSRMMD and other software programs for identifying genome-wide SSR feature loci.

Software	Thread	Rice (Zhenshan97, ∼0.39 Gb)			Cotton (TM1, ∼2.29 Gb)			Wheat (CS, ∼14.23 Gb)		
		Number	Time (m:s)	Mem (Mb)	Number	Time (m:s)	Mem (Mb)	Number	Time (m:s)	Mem (Mb)
SSRIT	1	111,960	5:53	131.55	384,488	34:45	343.04	1,345,128	210:47	2,503.85
MISA	1	111,905	5:50	205.52	384,400	35:31	382.60	1,343,830	212:45	4,195.61
GMATA^*a*^	1	111,905	7:28	85.48	384,400	42:15	361.72	1,343,831	254:46	1,739.50
PERF	1	111,960	8:49	211.60	384,488	52:32	522.91	1,345,128	320:36	3,325.42
Kmer-SSR	1	111,960	14:08	123.05	384,488	83:14	169.00	1,345,128	516:19	1,028.44
Kmer-SSR	12	111,960	5:28	321.95	384,488	28:20	353.96	1,345,128	205:19	1,251.40
SSRMMD	1	111,960	4:49	139.38	384,488	28:36	421.95	1,345,128	175:19	2,404.68
SSRMMD	6	111,960	1:08	466.19	384,488	6:46	1,044.88	1,345,128	43:40	5,972.30
SSRMMD	12	111,960	0:49	731.02	384,488	4:28	1,717.92	1,345,128	27:05	11,248.89

*Note. SSRMMD, Simple Sequence Repeat Molecular Marker Developer; SSR, simple sequence repeat. ^*a*^Because GMATA and Kmer-SSR could not simultaneously mine different types of motifs in one task, these two programs were multiply performed to identify SSRs, then the time was added, and memory peak was selected as the maximum among all tasks.*

### Verification of the Performance of SSRMMD to Mine Polymorphic Simple Sequence Repeats

CandiSSR and GMATA were compared with SSRMMD. First, compared with CandiSSR, SSRMMD mined approximately doubled the number of polymorphic SSRs, and CandiSSR discarded numerous monomorphic SSRs from among these candidate markers (Table 3). Second, compared with GMATA, SSRMMD mined more polymorphic SSRs in rice and cotton, but less in wheat. However, because GMATA identified polymorphisms through e-PCR amplification products, which yield two forms of false positives, (1) the target SSR did not exist in the product and (2) the target SSR in the product was the same size as the reference SSR. Hence, we generated a script^3^ to rectify the output of GMATA, and we found that the actual polymorphic SSR numbers of GMATA were 7,452 (rice), 21,675 (cotton), and 188,342 (wheat). GMATA had a high false-positive rate, being 36.86% (4,350 of 11,802), 30.06% (9,317 of 30,992), and 25.33% (63,902 of 252,244) for rice, cotton, and wheat, respectively, thus implying a defect in the GMATA pipeline. SSRMMD required markedly less time, especially in the wheat genome (Table 3). Unfortunately, we could not quantify memory consumption because GMATA and CandiSSR called numerous other programs and scripts for mining polymorphic SSRs. Furthermore, we compared the output of polymorphic SSRs between SSRMMD and two other software programs. As shown in Figures 3D–F, approximately 70.48% (rice), 37.11% (cotton), and 49.19% (wheat) of the SSRMMD outputs were novel in comparison with other two software programs.

**TABLE 3:** Performance comparison between SSRMMD and other two software programs for developing candidate polymorphic SSRs.

Organism	Rice (∼0.39 Gb)			Cotton (∼2.29 Gb)			Wheat (∼14.23 Gb)		
Software	SSRMMD	GMATA	CandiSSR^*c*^	SSRMMD	GMATA	CandiSSR	SSRMMD	GMATA	CandiSSR^*d*^
Total number of SSR^*a*^	111,960	111,905	111,905	384,488	384,400	384,400	1,345,128	1,343,831	1,331,146
Number of candidate marker	68,242	34,667	8,230	292,307	166,813	16,315	572,023	477,531	129,461
Candidate marker rate (%)	60.95	30.98	7.35	76.02	43.40	4.24	42.53	35.54	9.73
Number of monomorphic SSR	50,895	22,865	0	259,972	135,821	0	364,279	225,287	0
Number of polymorphic SSR	17,347	11,802	8,230	32,335	30,992	16,315	207,744	252,244	129,461
Polymorphic rate (%)	15.49	10.55	7.35	8.41	8.06	4.24	15.44	18.77	9.73
Time (min:s)^*b*^	6:08	16:15	8,117:37	30:30	119:06	1,746:23	184:55	6,363:45	118,953:53

*Note. SSRMMD, Simple Sequence Repeat Molecular Marker Developer; SSR, simple sequence repeat. ^*a*^Total number of SSR referred to Zhenshan97 (rice), TM1 (cotton), and CS (wheat). ^*b*^Because GMATA could not simultaneously mine different types of motifs in one task, it was multiply performed to identify SSRs, and then the time was added. ^*c*^Because CandiSSR could not normally calculate chromosome 8 of rice (the program stopped at the BLAST stage), the results of CandiSSR did not include chromosome 8. ^*d*^Because CandiSSR spent numerous time to mine polymorphic SSRs of wheat, we only selected chromosome 5D (closest to the total SSR density of wheat) to estimate the number of polymorphic SSRs.*

### Experimental Verification of the Accuracy of Polymorphic Simple Sequence Repeats

**FIGURE 4:**
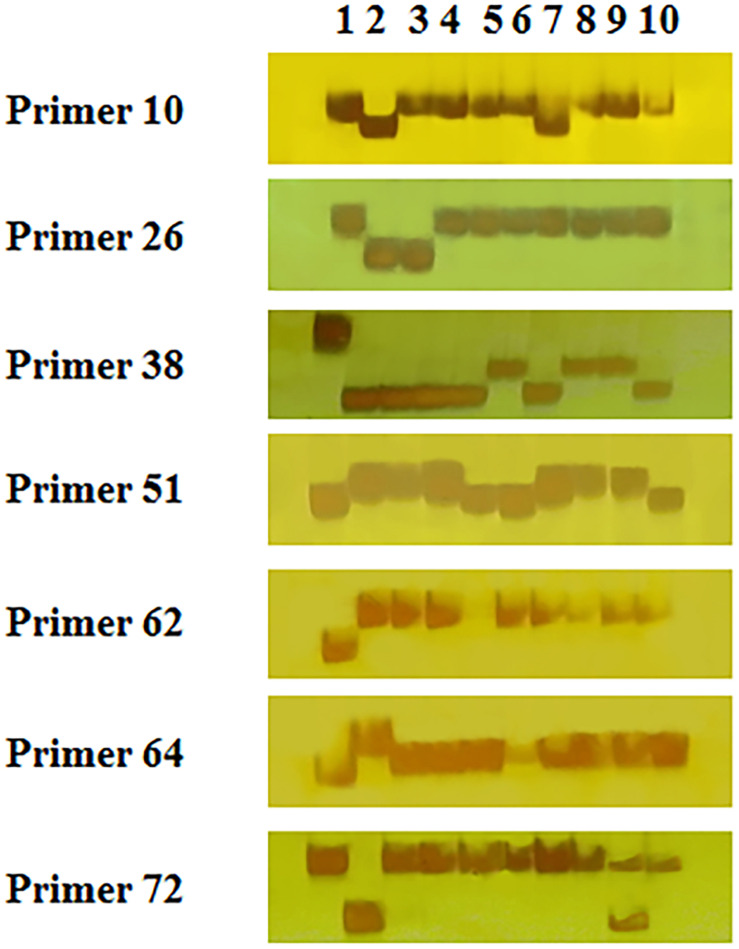
Experimental validation of seven randomly selected polymorphic simple sequence repeats (SSRs) in 10 wheat genotypes. Lanes 1–10 were PCR products of CS, AK58, CM107, CN16, MM37, ZM012542, ZM000652, ZM018703, ZM003222, and ZM003284, respectively.

To verify the accuracy of the output by SSRMMD, 80 pairs of polymorphic SSRs were randomly selected to identify polymorphisms in 10 wheat cultivars. As shown in Figure 4 and Supplementary Table 1, 56 independent products were successfully amplified using 56 primer pairs. However, the remaining 24 primer pairs did not yield stable or clear bands, and the reasons may include the following: (1) we did not optimize the PCR amplification conditions for each primer pair and used a uniform annealing temperature for all primer amplifications; and (2) some primer designs were created in batches generated under uniform conditions, which may have defects. Forty-four (∼79%) among these 56 primer pairs revealed polymorphisms in CS and AK58, suggesting that SSRMMD had a high accuracy.

## Discussion

With rapid innovations in sequencing technologies, third-generation DNA markers such as single-nucleotide polymorphisms have become widely used (Zhou Z. et al., 2015; Yang et al., 2017). However, SSRs are still used in various genetic studies such as quantitative trait loci mapping, genotyping, genetic diversity, and marker-assisted selection because of their codominant inheritance, multi-allelic nature, and ease of amplification *via* PCR operation (Varshney et al., 2005; Ramu et al., 2009; Kaur et al., 2015). These features are not applicable to single-nucleotide polymorphisms. Therefore, development of SSR markers from diverse organisms still is important in biological studies.

*In vitro* SSR marker development based on the creation of a genomic library, screening of positive clones, and subsequent sequencing is time-consuming and expensive. Song et al. (2005) only developed 540 SSR flanking primer pairs in the wheat mapping study by *in vitro* methods. However, we easily obtained millions of SSR loci from the wheat CS genome using our *in silico* methods (Table 1). Undoubtedly, it is more rapid and economical to develop SSR markers by using bioinformatics tools and genotypic data, and *in silico* methods have gradually replaced *in vitro* methods.

Although numerous software programs have been developed for mining perfect SSRs from assembled sequences, the accuracy, speed, and flexibility of these programs need to be balanced to suit the users’ needs. SSRIT can completely mine SSRs (Table 2); however, when used only for mining a certain motif, such as tetra-nucleotide and hexa-nucleotide motifs for rice (Zhenshan97), SSRIT displayed 82.94% and 87.36% error rates, respectively (data not shown), implying a defect in the algorithm of SSRIT. In contrast, MISA and GMATA were inadequate for SSR mining. Although Kmer-SSR supported multi-threading, this support was inadequate, and this program can only be run on Linux. Furthermore, GMATA and Kmer-SSR had inflexible motif thresholds; these two programs needed to be performed in multiple tasks to identify SSRs. PERF was inflexible owing to its dependence on other modules (Table 1), and the computational speed was highly dependent on the motif thresholds, thus displaying a poor performance in the present tests. However, SSRMMD displayed an adequate performance in all aspects. SSRMMD completely mined credible SSRs (Figures 3A–C); furthermore, SSRMMD was rapid, especially for large genomes (Table 2); moreover, SSRMMD was flexible and did not rely on additional modules and could theoretically be run on any machine with PERL5 installed (Table 1).

The ever-increasing availability of plant and animal genomes and transcriptomes (Kersey et al., 2017; Marschall et al., 2018) has resulted in large data resources for developing polymorphic SSRs. In the past 3 years, certain software programs were reported for this purpose; however, they were all based on a complex pipeline and utilize numerous other software programs, increasing their dependence and decreasing their portability. For example, CandiSSR called numerous other programs during development, including MISA, Primer3, BLAST, and ClustalW (Table 1), among which BLAST was the most prominent reason for its low computational speed. Furthermore, the formatdb program in BLAST could not build an entire wheat genome library; hence, to complete the assessment, we artificially modified the source code of CandiSSR to enable it to normally perform computations with wheat. Similarly, GMATA depended on other programs when developing polymorphic SSRs, including e-PCR and Primer3. However, our proposed SSRMMD did not have these limitations, and SSRMMD used a stringent algorithm to assess the conservativeness and uniqueness of SSR flanking sequences. Performance assessments revealed that SSRMMD identified more novel polymorphic SSRs at extremely high speed (Table 3 and Figures 3D–F).

Furthermore, we performed molecular biology assays for 80 randomly selected polymorphic SSRs of wheat to confirm the accuracy of SSRMMD, and we found that SSRMMD had an accuracy of up to 79% (Figure 4 and Supplementary Table 1). We further examined 24 pairs of SSRs not yielding stable or clear bands, and we found that 19 of them were developed through GMATA. Similarly, 7 of the 12 non-polymorphic SSRs were developed using GMATA (data not shown), indicating that these inaccurate results may have been obtained from the wheat genome itself.

Nonetheless, Gao et al. (2019) recently used SSRMMD to assess the barley genome in quantitative trait loci mapping study, and they reported that SSRMMD has an excellent algorithm for mining polymorphic SSRs.

## Conclusion

In this study, we proposed a rapid, accurate, and flexible algorithm named SSRMMD for mining perfect SSR loci and further mining candidate polymorphic SSRs in accordance with any size of assembled sequence. Our program can easily collect numerous polymorphic SSRs from genomes and transcriptomes of diverse organisms and will undoubtedly accelerate numerous types of genetic studies including those of quantitative trait loci mapping, genotyping, and genetic diversity.

## Data Availability Statement

All datasets presented in this study are included in the article/Supplementary Material.

## Ethics Statement

The experiments comply with the ethical standards in the country in which they were performed.

## Author Contributions

XG and HS conducted data analysis and drafted the manuscript. SY, ZW, CL, SL, and JM performed the validation experiment and helped with data analysis. GC helped to draft the manuscript. TL participated in the design of the study and partially revised the manuscript. YL designed and coordinated this study and revised the manuscript. All authors have read and approved the final manuscript.

## Conflict of Interest

The authors declare that the research was conducted in the absence of any commercial or financial relationships that could be construed as a potential conflict of interest.

## Footnotes

1 https://www.ncbi.nlm.nih.gov/

2 https://urgi.versailles.inra.fr/

3 https://github.com/GouXiangJian/CorrectGMATA

## Supplementary Material

TABLE S1 Molecular experiments to verify the accuracy of the output by SSRMMD in wheat.
